# Microbial Primer: The bacterial flagellum – how bacteria swim

**DOI:** 10.1099/mic.0.001406

**Published:** 2024-01-16

**Authors:** Judith P. Armitage

**Affiliations:** ^1^​ Department of Biochemistry, University of Oxford, South Parks Road, Oxford OX1 3QU, UK

**Keywords:** Brownian motion, chemotaxis, flagellum, motor, PMF, regulon, Reynold’s number, rotor, stator, T3SS

## Abstract

Bacteria swim using membrane-spanning, electrochemical gradient-powered motors that rotate semi-rigid helical filaments. This primer provides a brief overview of the basic synthesis, structure and operation of these nanomachines. Details and variations on the basic system can be found in suggested further reading.

## Physical constraints on swimming

Bacteria are so small that they experience almost no inertia, but high levels of viscosity, that is a world of low Reynold’s number. They do not displace liquid and when they stop there is no drift. This constrains the mechanism that can efficiently move them through the viscous environment. Bacterial cell bodies counter-rotate as the corkscrew-like semi-rigid filaments rotate, screwing their way through the medium. In addition, Brownian motion caused by the movement of surrounding molecules forces them off a straight path so they need to change direction every few seconds to keep moving on a favourable trajectory. They do this by transiently reversing the direction of rotation of the motor. Despite these constraints, bacteria can swim at speeds of up to 100 µm s^−1^, averaging 25 µm s^−1^, ~10–20 body lengths a second.

## Structure of the flagellum

Using his single-lensed microscope, van Leeuwenhoek in the 17th century first identified bacteria as living organisms because they moved rapidly and with apparent purpose. However, flagella are below the resolution of light microscopes, making them impossible to visualize on live cells, and the assumption until the 1960s was that they would be small versions of eukaryotic flagella. This is definitely not the case.

**Fig. 1.**
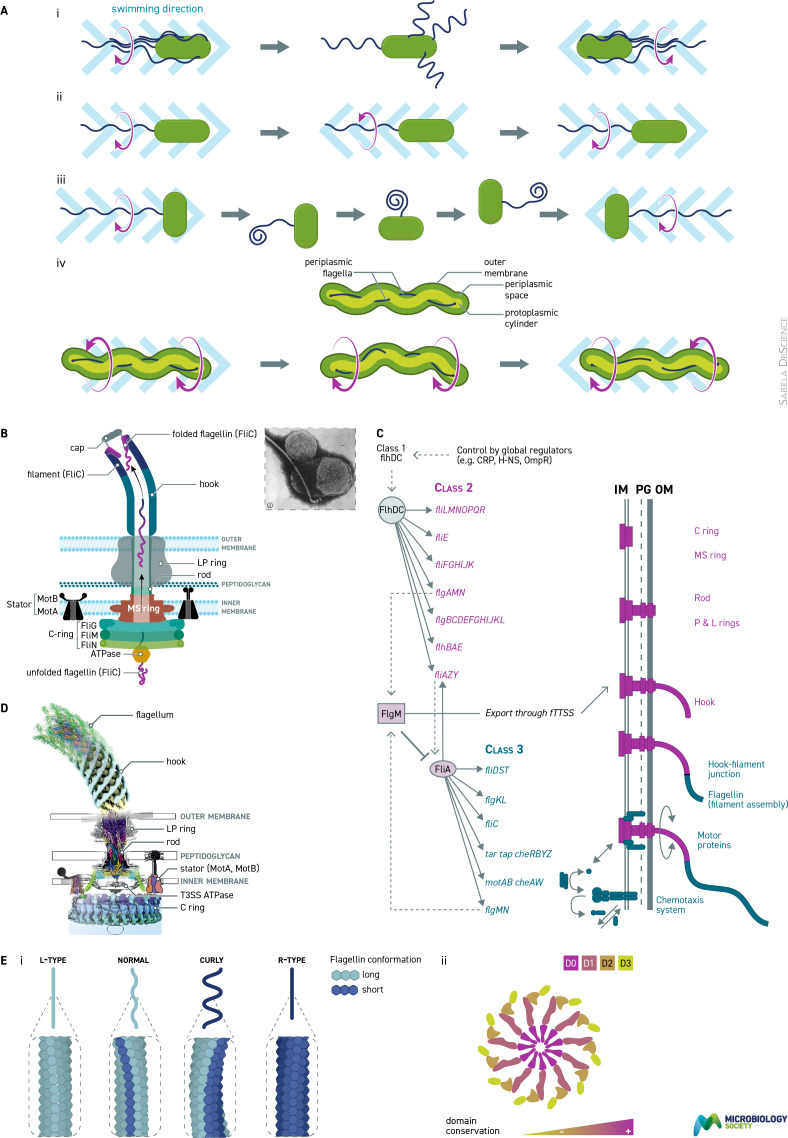
Structure and assembly of the bacterial flagellum. (a) Flagella patterns and associated swimming behaviour. (i) Peritrichous. Counter-clockwise motor rotation causes filaments to bundle and forward swimming, clockwise causes bundle to fly apart and cell to tumble. (ii) Polarly flagellate bacterium briefly reverses when direction of rotation changes. (iii) Unidirectional motor periodically stops and flagellum, without torque, relaxes and Brownian motion reorients until motor restarts and function filament reforms. (iv) Spiral species with polar tufts of filaments counter-rotating and periodically switching. Spirochete with internal filaments attached at each pole, smooth movement results from counter-rotation, rotating in the same direction, flexing of cell. (**b)** Cartoon of structure of the flagellar motor showing unfolded flagellin transported from cytoplasmic ATPase through the rod for polymerization at distal end of the filament. Associated C-ring is attached to MS ring and surrounded by stator complex. Small inset is a very early negative stain EM of a flagellar motor with hook and filament from plasmolysed cell. Reproduced with permission from [11]. (**c)** Genetic hierarchy showing sequential expression and assembly of the motor and the extracellular filament. Redrawn with permission from [12]. (**d)** Current detailed structure of the complete motor and flagellar system from cryo EM, cryo ET and crystal structures showing all components of the *Salmonella enterica* Typhimurium. One stator is shown engaged to peptigoglycan and one folded blocking ion channel. Image reproduced from [13]. (**e) **(i) Structural changes in the flagellin proteins of the filament causing changes in the wavelength caused by changes in the direction of rotation of the motor. 11-start helix formed by protofilaments has different potential interactions. (ii) Top view shows assembly of flagellin with conserved domains towards centre.

Different bacterial species can have very different numbers and positioning of flagella, from single polar through to tufts of polar flagella to all over the cell body, peritrichous, or even periplasmic (Fig. 1a). However, whatever the pattern of flagellation, the general structure and function of the flagellum are the same. This points to a very early origin in bacterial evolution, with some adaptation to life in specialist niches over time.

The flagellum spans from the cytoplasm to several μm outside the bacterial cell (with the exception of spirochetes, where the flagellum sits between the cytoplasmic and outer membranes). The most detailed studies of the structure of the motor are of *Escherichia coli* and *Salmonella enterica* Serovar Typhimurium. These related species have relatively simple motors with MS-ring (FliF) siting in the cytoplasmic membrane, below which is the key C-ring of 34 FliG proteins with an associated ring of FliM_1_/FliN_3_ switch proteins on the inner face. The MS-ring is attached to a helical rod protein complex that spans the periplasm, passing through the β-barrel porin-like L-P ring, which acts as a bushing. The rod is attached to the hook, which connects, via hook-associated proteins, to the filament itself (Fig. 1b). The C-ring is surrounded by a ring of stator proteins made up of 5 MotA, 2 MotB membrane-spanning proteins. MotBs attach the stators to the peptidoglycan and also form the ion channel.

The *E. coli* and *S. enterica* Typhimurium motors are made up of multiple copies of over 25 proteins, producing a structure ~45 nm in diameter and spanning the whole cell envelope and wall. Understanding the complete structure has taken over 50 years, starting with negative staining of isolated structures from developmental mutants through immunogold labelling to fluorescent labelling. Individual proteins and domains were crystalized, but complex integral membrane proteins such as the motor have been hard to analyse as intact structures. This changed with cryo electron microscopy and tomography (cryo-ET). Cryo-ET relies on structures being close to the membrane surface and cells being thin. The initial structure came from spirochetes or other small species with polar filaments or mini-cells of peritrichously flagellate species. Cryo-EM uses purification and *in vitro* averaging of very large numbers of isolated structures plus comparison of crystal structures. Together these approaches have allowed models to be developed that give us a very close approximation to the functional flagellar motor (Fig. 1d).

The stator complex has been even more difficult to characterize. Early genetic and biochemical crosslinking studies suggested 4 MotA and 2 MotB proteins per stator. Cryo EM has, however, established that the actual *E. coli* and *Salmonella* structure is 5 MotA surrounding 2 MotB proteins. The rotor and stator stoichiometry and structure does vary across species, as evolution has modified the composition to cope with different environments [1, 2] (see below).

The flagellar filament is composed of a polymer of ~20 000 copies of the protein flagellin. Flagellins vary across species but have common C and N terminal domains that fold into the centre of the filament and allow polymerization. If flagellins are purified by reducing the pH they will spontaneously repolymerize when neutralized. The variable domains are externally exposed and account for the antigenic properties of flagellin, often used for rapid diagnostic testing. Flagellins are exported through the rod and hook and polymerize distally into an 11-start helix, helped by hook-associated proteins. *E. coli* flagellin can fold into two conformations and this allows a polymer of a single protein to form a helix rather than a rod. The wavelength and handedness of the helix depends on how many of the 11 protofilaments of the filament are in a short versus long conformation, as this twists the structure. If all protofilaments are in the long or the shorter conformation the filaments are straight. The number of protofilament in different conformations is controlled by the link to the hook polymer and associated proteins and the force generated by the motor (Fig. 1e) [3, 4]. Some bacterial species have more than one flagellin forming similar, if slightly more complex, helices.

## Synthesis of the flagellum

The flagellum is probably the most complex structure in a bacterial cell and is energetically expensive to synthesize. As several components spontaneously polymerize it is important that this only happens at the right time and in the right place. Fifty or so genes are required to synthesize a flagellum, organized into a highly regulated series of operons, or regulon. A master regulatory operon ensures that new flagella are only synthesized at the right time for insertion into the right site (Fig. 1c) [5]. As different species may need to swim under different environmental conditions, the control of the master operon may differ. The master operon of *E. coli* and relatives is under the control of the cAMP-CRP system as *E. coli* does not synthesize flagella in high-glucose, high-nutrient environments. Alpha-proteobacteria, such as *Caulobacter crescentus*, control the operon with the CtrA protein linked to cell cycle. Whatever signal regulates the master operon, the expression leads to the sequential expression of subsequent operons, including a flagellum-specific sigma factor, σ^F^ or σ^28^. In *E. coli* class 2 operons are expressed and assemble the C-ring and rod in the cytoplasm membrane using the classical sec transport system. This is followed by formation, at the base of the C-ring, of an ATPase like transport system, the type 3 secretion system (T3SS), that is common to the flagellum and injectosome. The outer-membrane LP ring proteins are exported unfolded, and assembled through the Sec/Omp porin pathway.

The final sections of the flagellum expression and assembly pathway depend on the class 3 operons and are exported in strict sequence through the T3SS ATPase. After expression, the proteins, bound to a chaperone, bind the export gate and are exported unfolded up the centre of the rod and then the polymerizing hook. The proteins polymerize at the distal tip of the hook, aided by the hook-associated proteins. The hook size is regulated and once the correct length has been polymerized the flagellin proteins are expressed. These are expressed last because of control through an anti-sigma factor FlgM. FlgM prevents FliC, flagellin, being expressed until the hook has been synthesized. Once the hook is complete, FlgM is exported through the central pore and FliC is expressed. Again, FliC proteins move up the central pore of the growing flagellin filament for polymerization at the distal tip by the capping protein at the end of the filament. Transport of the unfolded proteins down the central filament channel appears passive and this may limit the length of the filament.

The stator proteins, along with chemotaxis proteins, are often encoded on operons separate from the core structural proteins, although often regulated by σ^28^. In almost all species, MotA and MotB are on the same operon and expressed together. They are not targeted to the newly formed motor. They are targeted to the membrane, where they form the MotA5 : MotB2 complex. The C-terminal end of MotB folds over the periplasmic face of the protein, blocking the ion channel, and the complex diffuses in the membrane until it encounters a rotor. Charge interactions between MotA and FliG appear to alter the conformation of MotB such that the C-terminal domain extends and binds the peptidoglycan of the cell wall. This causes the ion channel to open and allows rotation [6, 7].

## Motor mechanism

How do we know that the motor rotates? Eukaryotic flagella use a whiplash mechanism and microscopy of stained flagella shows a structure with the waveform damping along the length of the filament. A negatively stained or shadowed bacterial filament, however, shows an even wavelength down its full length, reminiscent of a rigid structure. Purified flagellin can be used to make specific antibodies, as can purified hook proteins. If a slide is coated with either hook or flagellin antibodies, bacteria then stick to the slide by a hook or a flagellum. The motor is still functional and, if it is attached by a single hook or flagellum, the bacterial cell body rotates smoothly around a central point, in the case of *E. coli*, switching direction of rotation briefly every few seconds. This strongly indicates a rotating motor not a whiplash movement.

We now know that the ring of FliG proteins is attached to the rod and thus the filament to the rotor. Rotation is driven as ions move through the MotB component of the MotAMotB stator. The ion driving force in most species is electrochemical proton gradient (pmf) generated across the membrane by aerobic or anaerobic respiratory electron transport, photosynthetic electron transport or, in the case of fermentative species, reverse proton flow through the ATP synthase [8]. Alkalophiles or species living in high-saline environments often use a sodium motive force rather than the (pmf) described above and the modified stators, PomA PomB, allow sodium rather than proton movement to drive rotation.

**Fig. 2.**
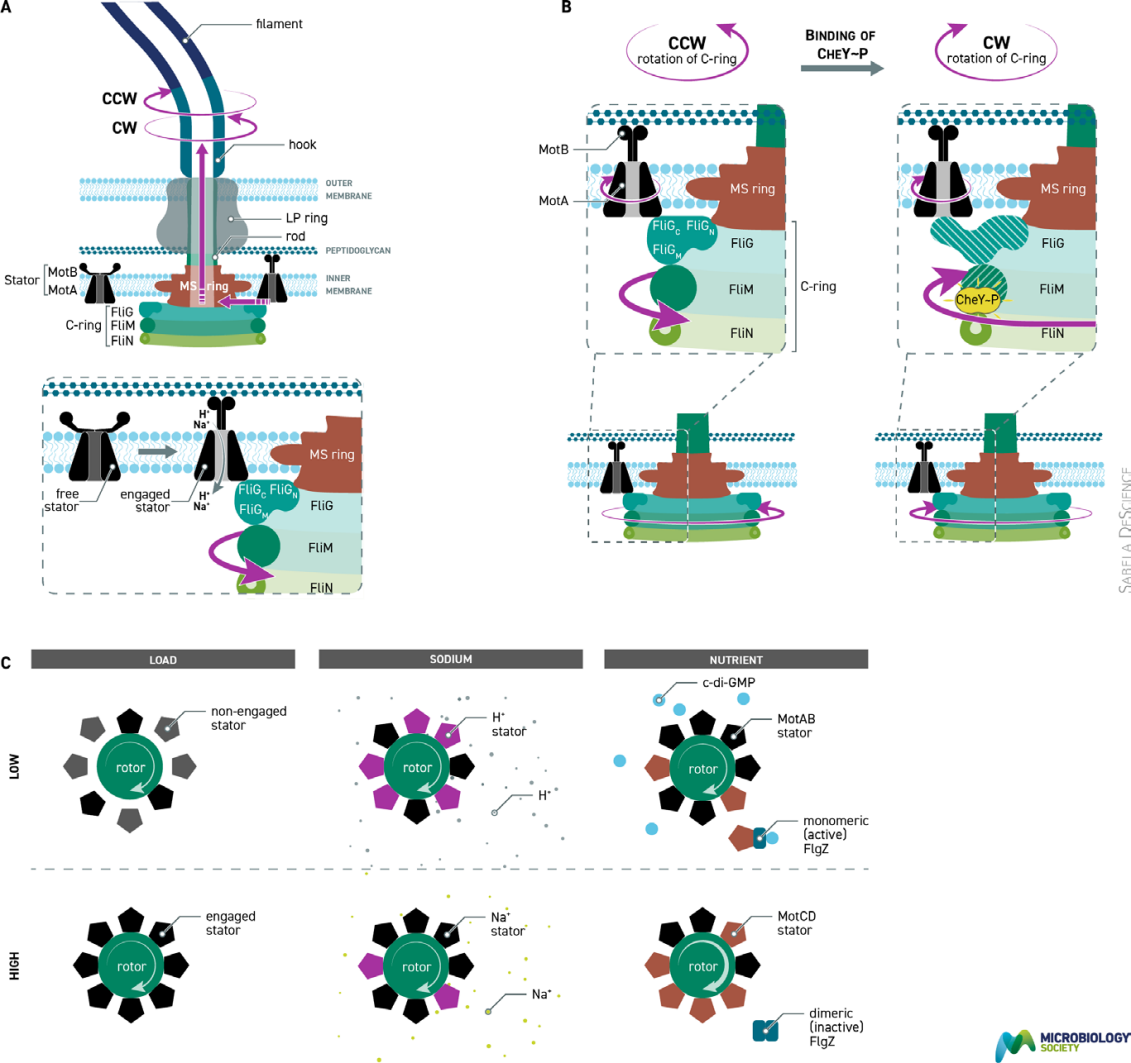
Molecular interactions within motor driving function. (**a)** Engagement of stators with rotor proteins driving rotation of rotor. (**b)** Spreading of conformational change of FliG on binding of chemotaxis signalling protein CheY~P to FliM, causing change in interaction with Mot proteins and switch in rotational direction. (**c)** Left: load increase in number of stators engaged with rotor as external viscosity increases. Middle: sodium change in number of sodium-driven vs proton-driven stators engaged around *S. oneidensis* rotor as sodium concentration changes. Right: nutrient swap from low- to high-load stators when *P. aeruginosa* encounters a surface is regulated by levels of c-di GMP, reflecting nutrient levels.

Once a stator has engaged the rotor and the peptidoglycan-binding domain of MotB is extended and bound, a structural change in MotB is thought to open the ion channel on MotB. Protons move through a specific path, engaging in *E. coli* with aspartate 32. It is thought that this interaction specifically changes MotA and the interaction with the charged surface of FliG, thus driving the rotation of both FliG and the stator, basically a two-cogwheel system driving rotation of the rotor. A proton-driven stator can rotate at ~300 r.p.s. and a sodium-driven motor at ~1300 r.p.s. The rotation of the rotor is transmitted through the associated rod to the hook and thus the filament, indeed the torque driven through the motor to the hook is thought to drive the conformation of the flagellin proteins and determine the number of protofilaments in the long and short form, and thus the helix and handedness [9] (Fig. 2a).

The rotors of most bacterial species switch direction of rotation periodically, with the frequency driven by signals from the chemosensory pathway. Associated on the cytoplasmic side of the FliG rotor ring is the FliM–FliN switch ring. CheY~P proteins from the chemosensory pathway transiently bind the switch proteins. When a critical number are bound this alters the average structure of FliG, changing the charge interaction, and the rotor switches rotational direction. This change in torque is transmitted through the hook to the associated proteins and thus the filament, with the force altering the structure of at least one protofilament to alter the handedness and wavelength of the filament, and this drives a change in swimming direction (Fig. 2b).

Using tethered cells and the expression of MotAB protein from plasmids in a *motAB* deletion, it was found that the speed of rotation of the tethered cell increased in approximately eight relatively even steps. This suggested not only that there are eight stators around a rotor, but that they can work independently of each other, each providing an even amount of torque. Later experiments showed that there can be a maximum of at least 11, but also surprisingly that this number is only found when the motor is having to produce high torque, either because it is driving rotation of the cell body rather than the filament or the cell is moving through very viscous environments. If the bacterium is swimming with a low load on the filament only one or two stators need to engage to drive swimming. However, as the external viscosity increases, the number of engaged stators increases to produce increased torque and allow continued swimming, until stall torque. This is the point at which the force output is not sufficient to overcome the external viscosity. If a cell is held at stall torque for ~10 min often rotation starts again, suggesting the addition of another stator and also suggesting that stators do not have fixed positions around the rotor. Indeed, we know that an individual stator only remains engaged for ~30 s before disengaging and diffusing in the membrane and possibly engaging with a distant rotor. There are ~200 free stators diffusing in the membrane. Engagement is therefore relatively weak and exchange is not the result of protein damage (Fig. 2c).

If the ion gradient is removed from a cell, the stators disengage and diffuse in the membrane, re-engaging once the proton gradient is formed, suggesting that the structural change occurring when ions flow through the MotB is required to keep MotB not only engaged with the rotor, via MotA, but also the peptidoglycan. Interestingly *Rhodobacter sphaeroides*, which has a stopping motor rather than a switching motor, keeps the stators engaged when stopped, even though the proton gradient is still present, suggesting a brake rather than clutch, although during a stop there is still an exchange of stator proteins.

The FliM and FliN switch proteins also exchange with pools in the cytoplasm, although the reasons remain unclear but may be linked to structural changes in the rotor and switching.

## Variations on a theme

Why do the stator proteins, in particular, exchange with pools. Clearly, structural changes in the rotor proteins keep stators engaged for longer when the extracellular forces increase, allowing increased torque production. In different species this is used for movement in very different environments [10]. *Pseudomonas aeruginosa* can swim in liquid, but can also use flagella to crawl through surface liquid. It encodes two sets of stator proteins, one for swimming under conditions of low load and a second that produces higher torque for moving on surfaces. However, a surface may have high or low levels of nutrient. If the nutrient levels are high, *P. aeruginosa* crawls on the surface, but if low environmental signals drive biofilm formation, motility is suppressed. Cyclic di-GMP binds a protein that interacts with the high torque stators, preventing engagement with the rotor and preventing swimming.

*Shewanella oneidensis* uses a sodium motive force and encodes sodium-driven stators. However, it also has genes for proton-driven stators, acquired by horizontal gene transfer (HGT), and these can engage if the sodium levels are very low, although swimming is not as efficient (Fig. 2c).

Many bacteria always live under conditions where they need a high torque motor, for example because they live in very viscous environments or the flagellum is rotated between the outer and inner membrane to corkscrew the cell body through the environment, as with spirochetes. These bacteria have all their stators engaged all the time. This allows cryo-EM structures with stators to be assembled. Some appear to have wider diameter rotor rings and it is speculated that this also allows more force to be generated. Other bacterial species have additional rings, in particular these are seen in species using sodium, but their roles are unclear.

Some bacteria can increase the number of flagella, depending on growth conditions, or express second flagella systems, probably acquired through HGT under different growth conditions, again to allow swimming on surfaces in the case of *Vibrio* spp. or in different environments in the case of *R. sphaeroides*. Studies on variability and the causes are, however, limited.

## Why swim?

Although not the topic of this primer, it is important to know that swimming has a purpose – to get bacteria to a better environment for growth or away from toxins. Synthesizing a flagellum is metabolically expensive and bacteria do not swim for fun. All motile bacteria control the switching or stopping frequency of the motor to bias the overall swimming direction towards an improving environment. Bacteria are too small to sense a gradient back to front; i.e. they have no nose. They sense the concentration of an effector and compare it with the concentration ~2 s ago. If the concentration is higher, direction changing is suppressed, if lower it increases. This biases the overall three-dimensional pattern of swimming in a favourable direction. This behaviour relies on chemosensory receptors that both signal and adapt, giving the system a memory. The bacterial chemosensory system regulating flagellar motor behaviour is sensitive to very small percentage changes in background concentration over four to five orders of magnitude, making it one of the most sensitive systems known. As might be expected, which effectors are sensed varies dramatically with the species and its favoured environment. Some species sense over 50 stimuli and others just 1 or 2.

While the flagellar system is conserved across all motile bacteria, the archael system is totally different, although both use the same chemosensory system.
